# Screening for Syphilis and Other Sexually Transmitted Infections in Pregnant Women — Guam, 2014

**DOI:** 10.15585/mmwr.mm6624a4

**Published:** 2017-06-23

**Authors:** Susan Cha, Tasneem Malik, Winston E. Abara, Mia S. DeSimone, Bernadette Schumann, Esther Mallada, Michael Klemme, Vince Aguon, Anne Marie Santos, Thomas A. Peterman, Gail Bolan, Mary L. Kamb

**Affiliations:** ^1^Epidemic Intelligence Service, Division of Scientific Education and Professional Development, CDC; ^2^Division of STD Prevention, CDC; ^3^Division of Viral Hepatitis, CDC; ^4^Division of Scientific Education and Professional Development, CDC; ^5^Emory University School of Medicine, Atlanta, Georgia; ^6^Guam Department of Public Health and Social Services; ^7^Guam Memorial Hospital Authority.

Prenatal screening and treatment for sexually transmitted infections (STIs) can prevent adverse perinatal outcomes. In Guam, the largest of the three U.S. territories in the Pacific, primary and secondary syphilis rates among women increased 473%, from 1.1 to 6.3 per 100,000 during 2009–2013 (*1*). In 2013, the first congenital syphilis case after no cases since 2008 was reported (*1*,*2*). Little is known about STI screening coverage and factors associated with inadequate screening among pregnant women in Guam. This study evaluated the prevalence of screening for syphilis, human immunodeficiency virus (HIV), chlamydia, and gonorrhea, and examined correlates of inadequate screening among pregnant women in Guam. Data came from the medical records of a randomly selected sample of mothers with live births in 2014 at a large public hospital. Bivariate analyses and multivariable models using Poisson regression were conducted to determine factors associated with inadequate screening for syphilis and other STIs. Although most (93.5%) women received syphilis screening during pregnancy, 26.8% were not screened sufficiently early to prevent adverse pregnancy outcomes. Many women were not screened for HIV infection (31.1%), chlamydia (25.3%), or gonorrhea (25.7%). Prenatal care and insurance were important factors affecting STI screening during pregnancy. Prenatal care providers play an important role in preventing congenital infections. Policies and programs increasing STI and HIV services for pregnant women and improved access to and use of prenatal care are essential for promoting healthy mothers and infants.

Syphilis, chlamydia, gonorrhea, and HIV infection in pregnant women can lead to mortality or severe morbidity in infants (*3*,*4*). Among pregnant women with untreated syphilis, an estimated 26% of pregnancies result in stillbirth and fetal loss, and another 12% in early neonatal death (*5*). Early prenatal screening and treatment is highly effective in reducing STI-related perinatal morbidity and mortality (*3*,*6*). The U.S. Preventive Services Task Force and CDC recommend screening all pregnant women for syphilis and HIV infection (*4*). Women should be screened for syphilis and HIV infection early in pregnancy at the first prenatal care visit to detect infections and initiate treatment before adverse outcomes occur (*3*). For syphilis, treatment in the first trimester and during the early second trimester can avert a majority of poor pregnancy outcomes caused by in utero infection (*6*). Women at increased risk for syphilis should be screened again at 28–32 weeks’ gestation and at delivery to detect new infections (*3*). Guamanian laws require health care providers to screen women for syphilis during pregnancy (10 GCA ^§^ 3323). Current U.S. recommendations also include screening for chlamydia and gonorrhea among pregnant women aged ≤24 years and older women at increased risk (*4*).

In 2014, approximately 73% of all births in Guam occurred at Guam Memorial Hospital. Among 2,478 live births at Guam Memorial Hospital during 2014, records of a random sample of 971 (39.2%) were reviewed to ascertain whether and when mothers were screened for STIs during pregnancy. One infant from each of five twin pairs was excluded, resulting in a total sample of 966 live births. Records with unknown conception date (23 records), test date (45), or those that were unclear about whether a syphilis test was done (33) were excluded. Thus, only records with information on the primary outcome of interest, gestational age at the time of syphilis screening, were included for analysis (865). Records with no missing information for HIV screening (842), chlamydia screening (870), or gonorrhea screening (873) were assessed in secondary analyses. It was assumed that all pregnant women should be screened for chlamydia because the prevalence is high in Guam (*1*). Because chlamydia and gonorrhea are usually tested together using a combination test, it was expected that most pregnant women would also be screened for gonorrhea. Thus, analyses for chlamydia or gonorrhea screening were not restricted to younger women. Standardized chart abstractions were performed at the hospital to obtain sociodemographic, clinical, and laboratory data. Medical records included information on prenatal care, STI screening during pregnancy, and pregnancy history. All data were deidentified for analyses. The study was exempt from review by the University of Guam Institutional Review Board.

The primary outcome, gestational age at the time of syphilis screening, was categorized as early screening through the early second trimester (up to 24 weeks’ gestation), late screening (after 24 weeks’ gestation), very late screening (after 32 weeks’ gestation), or no screening. Late screening after 24 weeks’ gestation includes very late screening. Screening for HIV, chlamydia, and gonorrhea were each categorized as “yes” or “no.” Potential correlates of STI screening included maternal age at delivery, race/ethnicity, education, employment status, insurance, gravidity, source of prenatal care, trimester of first prenatal care visit, and number of prenatal care visits.

Descriptive statistics were generated to assess the prevalence of inadequate syphilis screening by maternal characteristics. Separate Poisson regression models provided unadjusted and adjusted prevalence ratios (APR) and 95% confidence intervals (CIs) to determine factors associated with screening for syphilis, HIV, chlamydia, and gonorrhea during pregnancy. All nine potential correlates of screening were included in the multivariable Poisson regression models for fully adjusted estimates. All analyses were conducted using statistical software.

The majority (84%) of women in the sample population were aged 18–34 years, Chamorro (46%) or Chuukese (26%) ethnicity, unemployed (69%), uninsured or recipients of government-assisted health programs (i.e., Medicaid, Medically Indigent Program [MIP]) (70%), and multigravidas (70%) (Table 1). Approximately 90% of women had some source of prenatal care; 72.4% of women initiated prenatal care by the second trimester, and 73.3% had four or more prenatal care visits. However, 14.8% of women who initiated care in the first trimester and 10.7% who initiated care in the second trimester were initially screened for syphilis in subsequent trimesters or not at all. Overall, 577 (66.7%) women were first screened for syphilis early in pregnancy (up to 24 weeks’ gestation), 232 (26.8%) were screened late (after 24 weeks’ gestation), 110 (12.7%) were first screened very late in pregnancy (after 32 weeks’ gestation), and 56 (6.5%) had no screening (Table 1). Thus, approximately 33.3% did not have screening by 24 weeks’ gestation, and 19.2% had very late or no screening.

**TABLE 1:** Percentage and prevalence ratios of late or no syphilis screening among women who delivered at Guam Memorial Hospital, by maternal characteristics — Guam, 2014

Maternal characteristic (no. with available information)	Total no. (%) (N = 865)	% Late/No screening*(n = 288)	Unadjusted PR (95% CI)^†^	Adjusted PR (95% CI)^†,§^
**Age at delivery, yrs (865)**				
15–17	23 (2.7)	39.1	1.4 (0.7–3.0)	1.1 (0.5–2.5)
18–24	320 (37.0)	34.7	1.3 (0.8–1.9)	1.0 (0.6–1.8)
25–34	410 (47.4)	33.4	1.2 (0.8–1.8)	1.1 (0.7–1.8)
35–45	112 (12.9)	27.7	1.0	1.0
**Race/Ethnicity (862)**				
Chamorro	395 (45.8)	27.1	1.0	1.0
Chuukese	224 (26.0)	55.8	2.1 (1.6–2.7)^†^	0.9 (0.6–1.4)
Native Hawaiian and Other Pacific Islander^¶^	68 (7.9)	44.1	1.6 (1.1–2.4)^†^	0.8 (0.5–1.4)
Filipino	123 (14.3)	9.8	0.4 (0.2–0.7)^†^	0.5 (0.3–1.1)
Others	52 (6.0)	25.0	0.9 (0.4–1.6)	0.9 (0.4–1.8)
**Education (828)**				
<High school diploma	178 (21.5)	50.0	2.8 (1.9–4.1)^†^	1.1 (0.7–1.7)
High school diploma	452 (54.6)	34.3	1.9 (1.3–2.7)^†^	1.0 (0.7–1.6)
>High school diploma	198 (23.9)	18.2	1.0	1.0
**Employed (849)**				
Yes	265 (31.2)	15.5	1.0	1.0
No	584 (68.8)	41.4	2.7 (1.9–3.7)^†^	1.2 (0.8–1.8)
**Insurance (852)**				
None	135 (15.8)	57.8	4.4 (2.9–6.6)^†^	1.3 (0.7–2.2)
Medicaid	275 (32.2)	28.7	2.2 (1.5–3.3)^†^	0.8 (0.5–1.4)
MIP**	188 (22.1)	48.4	3.7 (2.5–5.5)^†^	1.1 (0.6–1.9)
Private	252 (29.6)	13.1	1.0	1.0
Other	2 (0.2)	100.0	7.6 (1.8–31.8)^†^	3.3 (0.7–14.7)
**Gravidity (576)**				
1	171 (29.7)	25.7	1.0	1.0
2	133 (23.1)	31.1	1.2 (0.9–1.7)	1.1 (0.7–1.6)
3	112 (19.4)	32.9	1.3 (0.9–1.9)	1.0 (0.6–1.6)
≥4	160 (27.8)	41.4	1.6 (1.2–2.2)^†^	1.2 (0.8–1.9)
**Source of PNC (849)**				
Public	361 (42.6)	36.3	2.1 (1.6–2.9)^†^	1.0 (0.7–1.5)
Private	383 (45.1)	17.0	1.0	1.0
Other	19 (2.2)	10.5	0.6 (0.2–2.5)	0.7 (0.1–5.1)
None	86 (10.1)	96.5	5.7 (4.1–7.9)^†^	1.5 (0.2–11.2)
**Trimester of first PNC visit (823)**				
1st	278 (33.8)	5.0	1.0	1.0
2nd	318 (38.6)	19.8	3.9 (2.2–7.0)^†^	2.0 (1.0–3.7)
3rd	138 (16.8)	73.2	14.5 (8.3–25.4)^†^	5.6 (2.9–10.6)^†^
No PNC	89 (10.8)	95.5	19.0 (10.8–33.4)^†^	4.0 (0.5–31.9)
**No. of PNC visits (834)**				
0–3	223 (26.7)	73.1	21.8 (9.7–49.3)^†^	4.6 (1.7–12.9)^†^
4–6	197 (23.6)	34.0	10.2 (4.4–23.4)^†^	4.0 (1.5–11.0)^†^
7–10	235 (28.2)	14.0	4.2 (1.8–10.0)^†^	2.4 (0.9–6.5)
≥11	179 (21.5)	3.4	1.0	1.0

**Abbreviations:** CI = confidence interval; MIP = medically indigent program; PNC = prenatal care; PR = prevalence ratio.

* Late screening after 24 weeks’ gestation includes very late screening.

^†^ Statistically significant.

^§^ Estimates adjusted for maternal age, race/ethnicity, education, employment, insurance, gravidity, source of prenatal care, prenatal care initiation, and number of visits.

^¶^ Other Pacific Islander groups include Kosraean, Marshallese, Palauan, Pohnpeian, and Yapese.

** MIP provides financial assistance for health care services to Guam residents not eligible for Medicaid or Medicare.

In bivariate analyses, inadequate syphilis screening was associated with race/ethnicity, education, employment, insurance, gravidity, and prenatal care initiation and number of prenatal care visits (Table 1). After adjusting for all nine screening correlates, late prenatal care and low number of prenatal care visits were significantly associated with late or no syphilis screening (Table 1). Women who began prenatal care in their third trimester had 5.6 times the prevalence of late or no syphilis screening compared with women who received care in their first trimester. Women with fewer prenatal care visits or no prenatal care had a four- to fivefold increase in prevalence of late or no syphilis screening compared with women with 11 or more prenatal care visits (Table 1). Similar results were observed when assessing correlates of very late or no syphilis screening.

Approximately 31.1% of women were not screened for HIV, 25.3% were not screened for chlamydia, and 25.7% were not screened for gonorrhea during pregnancy. In the multivariable regression model, insurance type, source of prenatal care, and fewer visits were significantly associated with lack of HIV screening (Table 2). Women with no insurance and those enrolled in Medicaid or MIP had twice the prevalence of not receiving HIV screening compared with privately insured women. Women with fewer than four prenatal care visits had 3.8 times the prevalence of no HIV screening compared with women with the currently recommended 11 or more prenatal care visits. Women who received prenatal care from a public clinic had a lower prevalence of no HIV screening than did those receiving care from private clinics (APR = 0.5, 95% CI = 0.3–0.7). Moreover, lack of screening for chlamydia or gonorrhea was associated with race/ethnicity, employment, insurance, source of prenatal care, prenatal care initiation, and number of prenatal care visits; however, differences were not statistically significant in fully adjusted models. Screening for chlamydia or gonorrhea did not differ across age groups.

**TABLE 2:** Prevalence ratios for lack of screening for HIV, chlamydia, or gonorrhea during pregnancy among women who delivered at Guam Memorial Hospital, by maternal characteristics — Guam, 2014

Maternal characteristic	HIV*^,†^		Chlamydia*^,§^		Gonorrhea*^,¶^	
	Unadjusted PR (95% CI)	Adjusted PR (95% CI)	Unadjusted PR (95% CI)	Adjusted PR (95% CI)	Unadjusted PR (95% CI)	Adjusted PR (95% CI)
**Age at delivery (yrs)**						
15–17	1.2 (0.5–2.8)	0.9 (0.3–2.2)	0.7 (0.2–1.9)	0.6 (0.2–2.0)	0.7 (0.2–1.9)	0.6 (0.2–1.9)
18–24	1.2 (0.8–1.7)	0.9 (0.5–1.5)	0.9 (0.6–1.4)	0.9 (0.5–1.6)	0.9 (0.6–1.4)	0.9 (0.5–1.7)
25–34	1.1 (0.7–1.5)	0.9 (0.6–1.4)	0.9 (0.6–1.3)	0.9 (0.6–1.5)	0.9 (0.6–1.3)	0.9 (0.6–1.5)
35–45	1.0	1.0	1.0	1.0	1.0	1.0
**Race/Ethnicity**						
Chamorro	1.0	1.0	1.0	1.0	1.0	1.0
Chuukese	1.2 (0.9–1.6)	0.8 (0.5–1.2)	1.9 (1.4–2.6)*	0.8 (0.5–1.4)	1.9 (1.4–2.5)*	0.8 (0.5–1.4)
Native Hawaiian and Other Pacific Islander**	0.9 (0.6–1.5)	0.7 (0.3–1.3)	1.1 (0.6–1.9)	0.8 (0.4–1.6)	1.1 (0.6–1.9)	0.8 (0.4–1.6)
Filipino	0.5 (0.3–0.8)*	0.8 (0.4–1.5)	0.8 (0.5–1.4)	1.0 (0.6–1.8)	0.9 (0.5–1.4)	1.0 (0.6–1.8)
Others	0.8 (0.5–1.4)	0.8 (0.4–1.8)	1.8 (1.1–3.0)*	1.2 (0.6–2.4)	1.9 (1.2–3.1)*	1.1 (0.5–2.3)
**Education**						
<High school diploma	2.6 (1.7–4.0)*	1.2 (0.7–2.1)	1.4 (1.0–2.1)	0.8 (0.5–1.3)	1.4 (0.9–2.0)	0.7 (0.4–1.2)
High school diploma	2.1 (1.4–3.1)*	1.2 (0.8–2.0)	1.1 (0.8–1.5)	0.7 (0.4–1.0)	1.0 (0.7–1.5)	0.6 (0.4–1.0)
>High school diploma	1.0	1.0	1.0	1.0	1.0	1.0
**Employed**						
Yes	1.0	1.0	1.0	1.0	1.0	1.0
No	1.8 (1.4–2.5)*	0.9 (0.6–1.4)	1.6 (1.1–2.2)*	1.0 (0.6–1.6)	1.6 (1.1–2.2)*	1.0 (0.6–1.6)
**Insurance**						
None	3.2 (2.1–4.9)*	2.3 (1.2–4.2)*	3.3(2.3–4.8)*	1.4 (0.7–2.7)	3.3 (2.2–4.8)*	1.4 (0.7–2.6)
Medicaid	2.8 (1.9–4.1)*	2.1 (1.3–3.6)*	1.1 (0.7–1.7)	0.8 (0.4–1.5)	1.1 (0.7–1.7)	0.8 (0.4–1.5)
MIP^††^	2.5 (1.7–3.9)*	2.4 (1.2–4.4)*	1.6 (1.0–2.4)	1.0 (0.5–2.1)	1.5 (1.0–2.3)	1.0 (0.5–2.0)
Private	1.0	1.0	1.0	1.0	1.0	1.0
Other	7.4 (1.8–30.6)*	10.1(2.1–5.4)*	0.0	0.0	0.0	0.0
**Gravidity**						
1	1.0	1.0	1.0	1.0	1.0	1.0
2	1.3 (0.9–1.8)	1.0 (0.7–1.6)	1.1 (0.8–1.6)	0.8 (0.5–1.3)	1.0 (0.7–1.5)	0.8 (0.5–1.3)
3	1.4 (0.9–2.0)	1.1 (0.7–1.8)	1.1 (0.8–1.8)	0.8 (0.5–1.4)	1.1 (0.8–1.7)	0.8 (0.5–1.4)
≥4	1.4 (1.0–2.0)	0.9 (0.6–1.5)	1.2 (0.8–1.7)	0.8 (0.5–1.3)	1.0 (0.7–1.5)	0.8 (0.4–1.3)
**Source of PNC**						
Public	0.9 (0.6–1.2)	0.5 (0.3–0.7)*	0.9 (0.6–1.3)	0.8 (0.5–1.4)	0.9 (0.6–1.3)	0.8 (0.5–1.4)
Private	1.0	1.0	1.0	1.0	1.0	1.0
Other	1.0 (0.4–2.6)	1.1 (0.4 –3.7)	0.3 (0.0–2.3)	0.6 (0.1–4.1)	0.3 (0.0–2.2)	0.6 (0.1–4.3)
None	4.2 (3.1–5.6)*	0.7 (0.2–3.2)	6.4 (4.6–8.9)*	1.2 (0.2–9.6)	6.2 (4.5–8.5)*	1.4(0.2–10.6)
**Trimester of first PNC visit**						
1st	1.0	1.0	1.0	1.0	1.0	1.0
2nd	1.6 (1.0–2.4)	1.0 (0.6–1.5)	1.1 (0.7–1.7)	1.1 (0.6–2.1)	1.1 (0.7–1.8)	1.1 (0.6–2.1)
3rd	2.2 (1.4– 3.4)*	1.0 (0.5–1.7)	1.7 (1.0–2.8)	1.4 (0.7–2.9)	2.0 (1.2–3.3)*	1.6 (0.8 –3.3)
No PNC	7.2(4.9–10.5)*	2.2 (0.5–9.6)	8.9(6.0–13.4)*	5.5 (0–44.0)	9.2 (6.2–13.9)*	5.6(0.7–44.6)
**No. of PNC visits**						
0–3	6.6(4.0–10.9)*	3.8 (1.8–7.9)*	4.1(2.7–6.4)*	1.5 (0.6–3.4)	4.4 (2.8–6.8)*	1.5(0.6– 3.4)
4–6	2.9 (1.7–5.0)*	3.1 (1.6–5.9)*	1.1 (0.6 –1.8)	1.2 (0.6–2.5)	1.2 (0.7–2.1)	1.3 (0.6–2.8)
7–10	1.4 (0.8–2.5)	1.4 (0.7–2.7)	0.8 (0.4–1.4)	0.8 (0.4–1.6)	0.8 (0.4–1.5)	0.8 (0.4–1.7)
≥11	1.0	1.0	1.0	1.0	1.0	1.0

**Abbreviations:** CI = confidence interval; HIV = human immunodeficiency virus; MIP = medically indigent program; PNC = prenatal care; PR = prevalence ratio.

* Statistically significant.

^†^ Restricted to records with no missing information on HIV screening (n = 842).

^§^ Restricted to records with no missing information on chlamydia screening (n = 870).

^¶^ Restricted to records with no missing information on gonorrhea screening (n = 873).

** Other Pacific Islander groups include Kosraean, Marshallese, Palauan, Pohnpeian, and Yapese.

^††^ MIP provides financial assistance for health care services to Guam residents not eligible for Medicaid or Medicare.

## Discussion

During a time of substantial increases in syphilis infection among women in Guam (*1*), one in three pregnant women in Guam were not screened for syphilis sufficiently early to prevent adverse pregnancy outcomes. Although no congenital syphilis was reported for Guam in 2014, another case occurred in 2015 for a rate of 30.4 per 100,000 live births (*1*). Risk factors for inadequate syphilis screening included delayed initiation of first prenatal visit and low frequency of prenatal visits; however, even women with multiple prenatal care visits did not always have adequate syphilis screening. Many women were also not screened for HIV infection, chlamydia, and gonorrhea during pregnancy, including those with multiple prenatal care visits. Those with government-assisted health care or no insurance and low number of prenatal care visits had a high prevalence of no HIV screening.

Because national data are not collected, relatively few studies have assessed prenatal STI screening in the United States, and available studies have had varying results. One study assessing a stratified random sample of birth records in eight U.S. states during 1998–1999 found that few women (1.7%) had no documented prenatal care, and there was a high rate of prenatal screening for syphilis (98.2%) but low screening for HIV infection (57.2%) (*7*). HIV screening was more common among women with Medicaid payment, blacks, and women aged <20 years. Another study, using 2009–2010 administrative claims data for women who received prenatal care in multiple U.S. states, reported high prenatal screening for syphilis (96.3%–97.8%) and lower screening for HIV infection (82.4%–85.4%), chlamydia (70.3%–83.1%), and gonorrhea (68.6%–74.8%); however, prenatal screening for specific STIs was similar among Medicaid and commercially insured women (*8*). This finding was in contrast to the data from Guam which indicated that more women covered by Medicaid or MIP had late or no syphilis screening (28.7%–48.4%) compared with privately insured women (13.1%).

Access to and use of quality prenatal care are essential for healthy pregnancies. A recent World Health Organization study estimated that 80% of adverse pregnancy outcomes caused by syphilis in 2012 occurred in women who received prenatal care. However, many did not receive recommended early screening and treatment, suggesting that multiple perinatal deaths and complications could have been prevented with appropriate adherence to recommendations (*9*). In the current study, 72.4% of women had initiated prenatal care by their second trimester, but overall, 26.8% had delayed syphilis screening and 6.5% had no screening. Approximately 10% of women lacked prenatal care, a higher percentage than women in the U.S. states (*10*). However, these results indicate substantial missed opportunities for screening because 15% of women who initiated care in their first trimester and 11% in their second trimester were first screened for syphilis in subsequent trimesters or not at all.

The findings in this report are subject to at least five limitations. First findings from Guam might not be generalizable to other U.S. territories or Pacific Island nations or to the 50 U.S. states. Nonetheless, the current study addresses an important issue in a population that has been largely underrepresented in extant literature. Second, live births from other facilities were not included, but Guam Memorial Hospital delivers most births in Guam. Third, some medical records had limited information on prenatal STI screening; however, multiple data sources were used to ensure completeness of data (e.g., laboratory reports, prenatal care records, labor and delivery charts). Fourth, because of the small sample size, gestational age at the time of syphilis screening could not be assessed in multiple categories (e.g., trimester of screening). Finally, because relatively few studies have evaluated STI screening among women, determining which variables are confounders or intermediates that would explain attenuated differences in fully adjusted models was difficult.

Many women were screened late or not at all for syphilis and other STIs during pregnancy. The U.S. rate of reported congenital syphilis has been increasing in tandem with increases in primary and secondary syphilis rates among women (*1*). This finding underscores the urgent need to screen all pregnant women and strengthen programs that provide STI, HIV, and perinatal services, particularly in areas like Guam, where resources are limited. Prenatal care providers and other health care workers play an important role in preventing congenital infections through routine early screening, treatment, and follow-up care. Further exploring strategies that improve prenatal screening practices in the health care system (e.g., provider education, standing orders, and opt-out testing) is essential for addressing challenges with screening adherence, even in areas with mandated screening laws. Policies and programs that support and improve use of quality prenatal care with early STI screening, particularly among women with limited resources or access to care, are essential for promoting healthy mothers and infants.

**Summary**

**What is already known about this topic?**

Current screening guidelines recommend early prenatal screening for syphilis and other sexually transmitted infections (STIs), because untreated infections can lead to adverse perinatal outcomes. In areas with increasing and high STI-related morbidity like Guam, prenatal screening coverage for STIs, and correlates of inadequate screening are not well understood.

**What is added by this report?**

Although the majority of pregnant women received prenatal care in Guam, nearly one third were not screened for syphilis and HIV infection, and one quarter were not screened for chlamydia and gonorrhea, as recommended. Few or no prenatal care visits, lack of insurance, and public insurance were associated with inadequate STI screening during pregnancy.

**What are the implications for public health practice?**

Prenatal care providers play an important role in perinatal STI prevention through early and routine screening for infections and providing appropriate treatment and follow up care. Policies and programs that increase STI and HIV services among pregnant women and improve access to and use of early prenatal care are essential for promoting healthy mothers and infants.
